# Comparison of hand, rotary, reciprocating and self-adjusting files in cleaning mesio-buccal canals of maxillary first molars

**DOI:** 10.6026/973206300200744

**Published:** 2024-07-31

**Authors:** Sainath Adsare, Ayesha Tehreem, Sahithi V., Ashwija Shetty, Karuna R. Siraparapu, Lipika Jain

**Affiliations:** 1 Department of conservative dentistry and Endodontics, The Oxford dental college Bangalore, Karnataka, India; 2 Department of conservative dentistry and Endodontics, HKE's Sri Nijalingappa Institute of Dental Sciences and Research, Gulbarga, Karnataka, India; 3 Department of conservative dentistry and Endodontics, Malla Reddy Dental College for Women Hyderabad, India

**Keywords:** Hand files, Rotary file, Self-Adjusting Files, Wave One

## Abstract

The smear layer removal capability of different instrumentation techniques on the mesio-buccal root canals of maxillary first molars is of interest to dentists. Sixty extracted maxillary first molars with fully developed apices, curved root canals, and curvatures between 30 to 45 degrees were selected. The teeth were divided into four groups (n=15) based on the instrumentation technique used. The samples were analyzed using a thermal field emission scanning electron microscope to assess smear layer removal at the apical, middle, and coronal thirds of the canals. The Wave One file system showed superior performance in smear layer removal across all three regions of the canal compared to hand files, Hyflex CM rotary files and SAF.

## Background:

Endodontic treatment aims to eliminate microorganisms from the root canal and prevent re-infection by thoroughly cleaning and shaping the canals using mechanical instrumentation, irrigants, and intracanal medicaments before obturation [1]. Removing this layer enhances dentinal tubule permeability, improving cleaning and the seal of the obturation material. Modern rotary nickel-titanium files, with their elasticity, facilitate cleaning and shaping, preventing issues like transportation and ledging common with stainless steel files [2]. These files effectively clean straight, narrow canals, but challenges remain with flat, oval-shaped, and curved canals found in certain teeth, such as molars and bicuspids [3]. Current rotary files, designed with spiral blades and helical formations, often fail to adequately prepare the buccal and lingual areas of flat root canals and the isthmus-facing sides of tear-shaped canals. This limitation can leave substantial untouched areas full of infected tissue and debris. Wu *et al.* [4] demonstrated similar issues with hand files, revealing that the technology may mislead operators into believing a canal is properly shaped, even when recesses remain contaminated. Such inadequacies can prevent proper obturation and sealing, providing spaces for bacterial growth and potential recontamination. Even in curved root canals, micro-CT scans show that rotary nickel-titanium files fail to consistently prepare all inner surfaces, as seen in studies of maxillary molars [5]. Improvements in rotary files, such as non-cutting tips and more flexible designs, have not fully resolved these issues [6, 7-8]. Consequently, a new concept, like the self-adjusting file (SAF), has been developed to address the inherent problems of traditional nickel-titanium instruments [3]. Therefore, it is of interest to compare the debridement effectiveness of hand (K-files), rotary (Hyflex CM), reciprocating (WaveOne), and SAF instruments in removing the smear layer from the mesio-buccal canals of maxillary first molars.

## Methodology:

This study was conducted in the Department of Conservative dentistry and Endodontics at H.K.E. Society's S. Nijalingappa Institute of Dental Sciences and Research, Gulbarga, Karnataka. Sixty extracted maxillary first molars were used for the research. Eligibility criteria for the study included maxillary first molars with fully developed apices, extracted for prosthetic or periodontal reasons, and teeth with curved root canals having curvatures between 30 to 45 degrees. Exclusion criteria were the presence of root caries or cracks, previous endodontic treatments, posts, cores, or crowns, teeth with open apices, calcified canals, or a mesiobuccal root with two canals.

## Sample preparation:

The teeth were cleaned of deposits using an ultrasonic scaler and stored in 10% formalin for up to six months. The teeth were then decoronated at the level of the cementoenamel junction (CEJ), and the mesiobuccal roots were separated. The mesiobuccal root canals were negotiated using#10 K-file, and an initial glide path was created with a #20 K-file. The working length was established, and root canals were prepared using the crown-down technique with Hyflex CM rotary files, WaveOne reciprocating files, and the Self Adjusting File (SAF) system.

## Division of samples into groups:

The samples were randomly divided into four groups (n=15):

[1] Group 1: Hand instrumentation with a step-back technique using stainless steel K-files up to ISO #30 file to working length.

[2] Group 2: Hyflex CM file, with cleaning and shaping using orifice enlargement with #25 (8%), followed by #20 (4%) and #25 (4%) to working length, and #20 (6%) for shaping the middle third.

[3] Group 3: WaveOne file, with cleaning and shaping using the primary file #25 (8%) to working length.

[4] Group 4: SAF, with cleaning and shaping using a single file (1.5mm diameter at the tip).

Irrigation was performed with 1 ml of 17% EDTA and 3 ml of 3% sodium hypochlorite after using each instrument.

## Preparation of the root canal:

Teeth were de-coronated at the CEJ level. ISO #10 K-files was inserted into the root canals until visible at the apical foramina under 4 x magnifications. Working lengths (WLs) were established by subtracting 1 mm from this point. Root canals were prepared with the crown-down technique for rotary file groups, following the manufacturer's instructions, with alternating irrigation using 3% sodium hypochlorite and 17% EDTA. All samples were rinsed with distilled water and dried with paper points. Sterilized cotton pellets were placed in the root canal orifices.

## Splitting and examination of teeth:

Longitudinal grooves were made on the buccal and lingual surfaces using a carbide disc without penetrating the canal. An osteotome was used to split the teeth along the grooves into two halves. The samples were gold-sputtered and sent for Scanning Electron Microscopy (SEM) analysis.

## SEM analysis:

In the thermal field emission SEM, samples were observed at the apical third (3 mm from the apex), middle third (7 mm from the apex), and coronal third (11 mm from the apex) by a double-blind test. Examiners scored the smear layer removal using the following criteria: 0: No smear layer or smear plugs. 1: No smear layer on the canal surface but mild smear plugs in dentinal tubules. 2: No smear layer on the canal surface but moderate smear plugs in dentinal tubules. 3: Moderate smear layer covering the canal surface with smear plugs. 4: Heavy smear layer completely covering the canal wall with smear plugs.

## Results:

The mean of smear layer analysis was calculated in each group at apical, middle, and coronal third and two-way ANOVA test was performed. The study compares the effectiveness of four instrumentation techniques (HAND, HYLEX, WAVE, and SAF) in removing the smear layer from root canals, with mean scores and standard deviations recorded for the apical, middle, and coronal regions. The HAND group consistently showed the highest mean scores (Apical: 3.80, Middle: 3.40, Coronal: 3.00), indicating the least effectiveness in smear layer removal. The WAVE group demonstrated the lowest mean scores across all regions (Apical: 2.33, Middle: 1.87, Coronal: 1.47), making it the most effective method. The SAF group showed intermediate performance (Apical: 3.33, Middle: 2.60, Coronal: 2.27), better than HYLEX but less effective than WAVE. HYLEX had moderate effectiveness (Apical: 3.53, Middle: 2.87, Coronal: 2.13), surpassing HAND but falling short compared to WAVE and SAF. Overall, the WAVE technique proved superior in smear layer removal, followed by SAF, HYLEX, and HAND (Table 1). The ANOVA results reveal significant differences in smear layer removal effectiveness across different instrumentation techniques and root canal regions. The intergroup analysis, with 3 degrees of freedom, shows a sum of squares of 3.4999, a mean square of 1.1666, and a high F-value of 52.98, indicating substantial variation among the techniques. Similarly, the between-regions analysis, with 2 degrees of freedom, has a sum of squares of 2.1278, a mean square of 1.0639, and an F-value of 48.32, demonstrating significant differences in smear layer removal across different canal regions. The error term, with 6 degrees of freedom and a mean square of 0.0220, indicates low variability within groups (Table 2). These high F-values suggest that both the instrumentation technique and the region of the root canal significantly affect the effectiveness of smear layer removal.

## Discussion:

Root canal treatment involves cleaning and shaping the root canals, placing an intra-canal dressing, and obturating the canal. One crucial factor for successful treatment is the seal created by the filling against the canal walls [9]. The role of the smear layer in this process has been debated. Some researchers suggest that maintaining the smear layer can block dentinal tubules and limit bacterial penetration by altering dentinal permeability, while others argue that it should be removed to avoid harboring bacteria and ensuring effective disinfection. The maxillary first molar, especially the mesio buccal (MB) root, presents challenges due to its complex anatomy. Thorough cleansing and disinfection of the canal system are essential for successful treatment, but no single irrigant can efficiently remove both the smear layer and organic debris. Hence, a combination of irrigants like sodium hypochlorite and EDTA is often used to enhance debridement [10]. Innovative approaches and materials have been developed to improve debridement. Ni-Ti rotary instruments have revolutionized root canal shaping, saving time and increasing debridement efficiency [11, 12]. These instruments have evolved to offer greater flexibility, increased fracture resistance, and better cutting efficiency [13, 14]. The WaveOne files, made with the M Wire NiTi alloy, use a reciprocating motion to effectively remove dentin [15, 16]. The Self-Adjusting File (SAF), which uses a back-and-forth motion and continuous irrigation, offers another approach [17]. Despite advancements, rotary and hand instrumentation techniques are still not completely effective in shaping all canal surfaces and irregularities. Studies show that the apical third of the canal often remains less clean than the middle and coronal thirds [18]. Sodium hypochlorite is known for its ability to dissolve organic debris and provide antimicrobial effects, while EDTA is effective in removing the smear layer. A combination of these irrigants is most effective, but overuse of EDTA can erode the canal walls. Therefore, a specific irrigation protocol is followed to balance efficacy and safety. Results from the study show that WaveOne files perform better in smear layer removal compared to hand files, Hyflex, and SAF, especially in the coronal, middle, and apical thirds of the canal. Root canal disinfection, critical for endodontic success, requires both mechanical preparation and irrigation. Mechanical disinfection is particularly challenging in oval or curved canals. The SAF's ability to adapt to canal walls is beneficial, but it is still less effective in the apical third. Larger canal diameters in the coronal and middle thirds allow better irrigant flow, improving smear layer removal. However, the apical third remains difficult to clean due to anatomical complexities and limited irrigant contact.

## Clinical significance:

Effective smear layer removal is crucial for preventing bacterial re-infection and ensuring long-term success in root canal treatments. The WaveOne file system offers a more reliable method for achieving cleaner canals, which is essential for optimal disinfection and sealing. This study supports the use of reciprocating file systems in clinical practice to improve endodontic outcomes, particularly in anatomically complex canals.

## Limitations of the study:

This study was limited by its small sample size and in vitro design, which may not fully replicate clinical conditions. Ensuring uniformity in tooth selection and canal curvature was challenging, potentially affecting result consistency. Only four instrumentation techniques were evaluated, possibly overlooking other effective systems. The subjective nature of smear layer assessment and the difficulties in cleaning the apical third highlight the need for further research. Additionally, the specific irrigation protocol used may not cover all effective combinations, and long-term clinical outcomes were not assessed.

## Conclusion:

All four instrumentation groups were able to remove the smear layer effectively but not completely, particularly in the apical third of the root canal. The WaveOne system demonstrated superior performance across all three regions. Cleaning and shaping were generally more effective in the coronal and middle thirds compared to the apical third for all systems.

## Figures and Tables

**Table 1:** Mean and standard deviation of all groups at three regions

	**HAND**		**HYLEX**		**WAVE**		**SAF**	
	**Mean**	**S.D**	**Mean**	**S.D**	**Mean**	**S.D**	**Mean**	**S.D**
Apical	3.8	0.4	3.53	0.49	2.33	0.47	3.33	0.59
Middle	3.4	0.49	2.87	0.49	1.87	0.49	2.6	0.49
Coronal	3	0.37	2.13	0.34	1.47	0.49	2.27	0.44

**Table 2:** Comparison of all groups at three regions with two-way ANOVA

	**D.F**	**Sum of Square**	**Mean sum of square**	**F Value**
Between groups	3	3.4999	1.1666	52.98
Between regions	2	2.1278	1.0639	48.32
Error	6	0.1321	0.022	
F value (p<0.05) shows significant difference between groups and between regions
